# Physical activity enjoyment and attitudes toward healthy nutrition in women: associations and demographic differences

**DOI:** 10.3389/fgwh.2026.1880236

**Published:** 2026-07-08

**Authors:** Bekir Erhan Orhan, Walaa Jumah Alkasasbeh, Aydın Karaçam, Umut Canlı, Adam Tawfiq Amawi

**Affiliations:** 1 Faculty of Sports Sciences, Istanbul Aydın University, Istanbul, Türkiye; 2 Department of Physical Education, Faculty of Sport Science, The University of Jordan, Amman, Jordan; 3 Faculty of Sports Sciences, Bandırma Onyedi Eylül University, Balıkesir, Türkiye; 4 Faculty of Sports Sciences, Tekirdağ Namık Kemal University, Tekirdağ, Türkiye; 5 Department of Movement Sciences and Sports Training, School of Sport Science, The University of Jordan, Amman, Jordan

**Keywords:** health promotion, healthy eating attitudes, lifestyle psychology, physical activity enjoyment, women's health behaviour

## Abstract

**Introduction:**

Women's physical activity and nutrition behaviours are key public health priorities, yet the affective and cognitive factors underlying these behaviours remain incompletely understood. This study examined the association between women's physical activity enjoyment and attitudes toward healthy nutrition and explored whether these constructs differed according to key sociodemographic and activity-related characteristics.

**Methods:**

A cross-sectional correlational study was conducted among 397 adult women (18–60 years; *M* = 31.43, SD = 10.97) living in Türkiye. Participants completed the Physical Activity Enjoyment Scale (PACES) and the Attitude Scale for Healthy Nutrition (ASHN) via an online questionnaire. Independent-samples *t*-tests, one-way ANOVA, Pearson correlation analyses, and simple linear regression were performed.

**Results:**

Physical activity enjoyment was positively associated with overall healthy nutrition attitudes (*r* = .28, *p* < .001). ASHN total scores significantly predicted PACES scores (*β* = .28), explaining 8.3% of the variance (R² = .083, *p* < .001). Married women reported significantly higher ASHN total scores than single women (*d* = .42, *p* < .001). Higher educational attainment and greater physical activity frequency were associated with more favourable healthy nutrition attitudes, while women engaging in physical activity ≥5 days/week reported the highest PACES scores. Athletic license status was associated only with the ASHN Emotion for Nutrition subscale (*p* = .04).

**Discussion:**

Physical activity enjoyment and healthy nutrition attitudes are modestly but consistently associated in adult women. These findings support integrated, subgroup-sensitive health promotion strategies that simultaneously target enjoyment of physical activity and positive nutrition attitudes to encourage healthier lifestyles.

## 1 Introduction

Women's health behaviours are a priority for public health because physical activity and nutrition patterns shape long-term physical and psychological outcomes across adulthood. Examining women specifically is warranted because gendered roles, constraints, and barriers can influence opportunities for sustained physical activity and healthy nutrition practices (1–3). A dynamic interplay of emotional, cognitive, and environmental factors shapes these behaviours. While physical activity and nutrition are widely recognized for their physiological benefits, the subjective experiences and psychological attitudes that underpin these behaviours are equally crucial in determining whether they are adopted and sustained over time (4–6).

Enjoyment is widely regarded as a central psychological determinant of sustained engagement in physical activity. Conceptually, enjoyment reflects a positive affective response experienced during or after an activity, including feelings of pleasure, satisfaction, and fulfilment (7–9). When physical activity is experienced as enjoyable, participation is more likely to be self-endorsed and intrinsically motivated-maintained because the behaviour is personally rewarding rather than performed primarily in response to external demands or obligations (10–13). In women, enjoyment may be shaped by both individual and contextual influences, including perceived competence, body image-related perceptions, social environment, and access to supportive and safe settings for participation (14–16). Over time, repeated positive affective experiences can strengthen the likelihood that physical activity becomes integrated into daily routines and sustained as a habitual health behaviour. In parallel, attitudes toward healthy eating represent an individual's evaluative stance toward food choices, dietary habits, and nutrition-related behaviours (17–19). These attitudes are multidimensional, involving beliefs, emotions, and behavioural intentions that are shaped by past experiences, cultural norms, health literacy, and self-perception (18, 20–22). A positive attitude toward healthy eating typically includes valuing nutritional quality, feeling confident in making informed choices, and associating healthy food with well-being and satisfaction (19, 21, 23). Conversely, negative or indifferent attitudes may lead to inconsistent dietary behaviours, emotional or convenience-driven eating, and a decreased focus on nutritional balance (17, 24, 25).

Although physical activity enjoyment and attitudes toward healthy nutrition are often studied independently, their potential interrelationship remains underexplored, particularly in adult women (26–28). Given that both constructs reflect affective and cognitive dimensions of health behaviour, it is plausible that individuals who derive pleasure from movement may also exhibit more favourable orientations toward nutrition (26, 29, 30). Such a link may reflect a broader health-conscious or self-care profile that extends across behaviours (30, 31). Importantly, these psychological tendencies are shaped by socio-cultural and environmental conditions, including access to resources, social support, lifestyle constraints, and exposure to health information (32, 33). As these influences vary across individuals, examining demographic diversity is essential for clarifying subgroup variability in women's health-related profiles (34, 35). In this study, age, education level, marital status, physical activity frequency, and athlete license were examined because they are commonly linked to health-related opportunities and constraints, including health literacy, access to resources, time availability, social support, and structured sport involvement, factors that may shape both enjoyment of physical activity and attitudes toward healthy nutrition (13, 36). Accordingly, this study contributes by integrating women's enjoyment of physical activity and their attitudes toward healthy nutrition within a single analytic framework and by examining demographic differences in these outcomes to provide a clearer, subgroup-sensitive picture of women's health behaviour profiles.

Therefore, this study aimed to examine the association between women's enjoyment of physical activity and attitudes toward healthy nutrition, and to describe whether these constructs differ across key sociodemographic and activity-related characteristics (age, education, marital status, athletic license status, and physical activity frequency).

On this basis, the following hypotheses were specified *a priori*. H1: Women's physical activity enjoyment is positively associated with their attitudes toward healthy nutrition, both at the ASHN total level and across its subdimensions (Information on Nutrition, Emotion for Nutrition, Positive Nutrition, and Malnutrition). H2: Attitudes toward healthy nutrition account for a statistically significant share of the variance in physical activity enjoyment. H3: Physical activity enjoyment and attitudes toward healthy nutrition differ across key sociodemographic and activity-related subgroups (age, education level, marital status, athletic-license status, and physical activity frequency). Because the design is cross-sectional, these hypotheses concern associations and group differences rather than causal effects.

## 2 Materials and methods

### 2.1 Study design

This study employed a cross-sectional, correlational design to examine the association between women's enjoyment of physical activity and their attitudes toward healthy nutrition and differences in these outcomes across demographic and activity-related characteristics (37). Because variables were measured at a single time point without manipulation, the findings describe naturally occurring relationships and do not support causal inference (38). Methodological and reporting principles appropriate to correlational research in behavioural sciences were followed (39). The study was reported in accordance with the STROBE statement for cross-sectional studies (40), and the completed checklist is provided as Supplementary File S1. Data collection occurred over approximately three months (April-June 2025).

### 2.2 Participants

Participants were recruited using convenience sampling via online dissemination (e.g., institutional networks and community-based sharing (37, 39)). Before starting the survey, individuals reviewed an electronic information page describing the study purpose and procedures, voluntary participation, confidentiality, and the right to withdraw at any time without penalty. Those who provided electronic written informed consent completed the online questionnaire, which required approximately 8–10 min. To protect data integrity, submissions were screened prior to analysis for duplicate entries and response patterns suggestive of inattentive or invalid responding; identified cases were removed. Data were collected between April and June 2025. Ethical approval was obtained from the Ethics Committee of the Faculty of Social and Human Sciences, Istanbul Aydın University (Decision No: 2025/3). Informed consent was captured electronically within the online survey as a mandatory consent item prior to accessing the questionnaire; this procedure was approved by the ethics committee. Because data collection was fully online, written consent was captured electronically rather than verbally.

Eligible participants were women aged 18 years or older, residing in Türkiye, who provided informed consent. Responses were excluded if eligibility criteria were not met or if questionnaires were incomplete. Participants self-reporting a medical condition restricting participation in physical activity were also excluded.

The final analytical sample comprised 397 adult women (mean age = 31.43 years). All participants were Turkish citizens, reflecting adult women within the socio-cultural context of Türkiye. Regarding education, 25.2% (*n* = 100) were high school graduates, 58.7% (*n* = 233) held a bachelor's degree, and 16.1% (*n* = 64) had a postgraduate degree. In total, 55.9% (*n* = 222) reported holding an athlete license, and 44.1% (*n* = 175) did not; 43.3% (*n* = 172) were married, and 56.7% (*n* = 225) were single (Table 1). An athlete's license indicates official registration with a recognised sports federation and participation in organised training and/or competitive sporting events. Population comparators for women in Türkiye are provided for context: median age = 35.2 years (2024) (41) and tertiary education attainment (≥25 years) = 22.7% (42); TurkStat reports marital status in four legal categories, so “single” is only interpretable if defined as “not married” (never married + divorced + widowed) (42).

**Table 1:** Characteristics of participants (*N* = 397).

Characteristic	Sample (*N* = 397)	Range	Population women 18 + in Türkiye
Marital status			TurkStat reports legal categories; binary coding not directly comparable
Married	172 (43.3%)	–	–
Single	225 (56.7%)	–	–
Athlete license			Not available/not directly comparable
Yes	222 (55.9%)	–	–
No	175 (44.1%)	–	–
Education level			Tertiary education attainment (women ≥25 y): 22.7% (2024)
High school	100 (25.2%)	–	–
Bachelor's degree	233 (58.7%)	–	–
Postgraduate degree	64 (16.1%)	–	–
Physical activity frequency (coded 1–4)	M = 2.38 (SD = 1.03)	1–4	Not available/not directly comparable
Age (years)	M = 31.43 (SD = 10.97)	18–60	Median age (women): 35.2 years (2024)

Physical activity frequency categories were: 0 days/week, 1–2 days/week, 3–4 days/week, and ≥5 days/week (coded 1–4); higher values indicate higher frequency. Population comparator values are provided where publicly available and are intended for contextual benchmarking rather than formal representativeness assessment; marital-status distributions are reported by TurkStat in legal categories and are not directly comparable to the study's binary coding. M, mean; SD, standard deviation; n, number of participants.

A sensitivity power analysis based on Fisher's z transformation indicated that with *N* = 397, the study had 80% power (two-tailed alpha = .05) to detect correlations of approximately *r* ≥ .14 (43).

### 2.3 Data collection tools

In this study, the Physical Activity Enjoyment Scale (PACES), adapted into Turkish by Özkurt et al. (44), and the Attitude Scale for Healthy Nutrition (ASHN), developed by Tekkurşun Demir and Cicioğlu (45), were used as measurement tools. Additionally, a brief study-specific personal information form captured demographic characteristics (age, marital status, education level, and athletic license status) and physical activity frequency via a single-item question. Data were collected via an online questionnaire platform. An English version of the survey guide (information/consent text, demographic items, and physical activity frequency item) is provided as Supplementary File S2. Scale descriptions and scoring procedures are provided as Supplementary File S3.

#### 2.3.1 Physical activity enjoyment scale (PACES)

The Physical Activity Enjoyment Scale (PACES) is a unidimensional measure of enjoyment experienced during physical activity. In the present study, the 8-item Turkish version adapted by Özkurt et al. (44) was utilised. Items are rated on a 7-point Likert scale (1 = Strongly Disagree to 7 = Strongly Agree) with no reverse-coded items; higher mean scores indicate greater physical activity enjoyment. The Turkish validation study supported a single-factor structure, explaining 76% of the total variance in exploratory factor analysis, and demonstrated good confirmatory model fit (*χ*²/df = 2.368, GFI = .98, CFI = .99, TLI = .99, RMSEA = .042, SRMR = .010).

#### 2.3.2 Attitude scale for healthy nutrition (ASHN)

The Attitude Scale for Healthy Nutrition (ASHN) was developed by Tekkurşun Demir and Cicioğlu (45). The scale consists of 21 items grouped under four factors: Information on Nutrition (IN; items 1–5), Emotion for Nutrition (EN; items 6–11), Positive Nutrition (PN; items 12–16), and Malnutrition (MP; items 17–21). The items are rated on a 5-point Likert scale with the response options “Strongly Disagree,” “Disagree,” “Neutral,” “Agree,” and “Strongly Agree.” Positive items are scored from 1 to 5, whereas negative items are reverse-scored from 5 to 1. Positive items include items 1–5 and 12–16; negative items include items 6–11 and 17–21. Two subdimensions warrant conceptual clarification because their items are reverse-scored. EN captures the affective pull toward energy-dense, palatable but nutritionally poor foods (e.g., pleasure derived from sugary, fried, and fast-food items); because these items are reverse-scored, a higher EN score reflects a weaker affective attachment to unhealthy foods rather than stronger positive emotion about nutrition *per se*. MP indexes the self-reported frequency of maladaptive eating behaviours (e.g., skipping main meals, daily consumption of junk food and carbonated drinks, eating on the go); as MP is likewise reverse-scored, a higher MP score denotes fewer maladaptive eating patterns. Consequently, for both subdimensions, and for the ASHN total, higher scores consistently index a more health-supportive nutritional orientation. The total score obtainable from the scale ranges from 21 to 105. Scores are interpreted as follows: 21 = very low, 23–42 = low, 43–63 = moderate, 64–84 = high, and 85–105 = ideally high attitude toward healthy nutrition. In the original validation, internal consistency (Cronbach's alpha) was.90 for IN,.84 for EN,.75 for PN, and.83 for MP. Confirmatory factor analysis indicated acceptable fit: *χ*²/df = 1.71, RMSEA = 0.04, PGFI = 0.74, PNFI = 0.82, GFI = 0.92, AGFI = 0.90, IFI = 0.98, NFI = 0.95, CFI = 0.98 (45).

#### 2.3.3 Physical activity level

Physical activity frequency was assessed to describe participants' habitual engagement in movement and to support subgroup comparisons, as participation frequency may influence physical activity enjoyment and relate to healthy nutrition attitudes. Physical activity level was measured using a single self-report item: “During the past 7 days, on how many days did you perform any physical activity (e.g., walking, running, sports, or exercise) for at least 30 min per day?” Responses were classified into four groups: sedentary (0 days), low activity (1–2 days/week), moderate activity (3–4 days/week), and high activity (≥5 days/week). This frequency-based categorisation is consistent with common public health and exercise-science practice for describing activity patterns and examining associations with health-related outcomes (46–48).

### 2.4 Statistical analysis

Prior to analysis, the dataset was screened for data-entry errors, missing values, outliers, distributional assumptions, and multicollinearity. No input errors were identified. All analyses were conducted using IBM SPSS Statistics (Version 25; IBM Corp., Armonk, NY, USA). Distributional assumptions were evaluated using the Shapiro–Wilk test and inspection of skewness and kurtosis. Independent-samples *t*-tests were used for two-group comparisons, and one-way analysis of variance (ANOVA) was applied for comparisons involving more than two groups. Effect sizes were reported for all group comparisons (Cohen's d for *t*-tests; eta squared [*η*^2^] for ANOVA). Associations between continuous variables were examined using Pearson's product-moment correlation coefficient. Simple linear regression was used to test whether ASHN total predicted PACES total. Because the focal aim was to quantify the bivariate association between the two constructs, this regression was specified as a simple (unadjusted) model; it does not control for potential confounders (e.g., age, marital status, education level, and physical activity frequency), so its coefficients are interpreted as descriptive associations rather than adjusted effects (see Limitations). Given that subgroup comparisons spanned five grouping variables and six outcomes, a deliberately conservative interpretive stance was adopted to limit inflation of the family-wise Type I error rate: omnibus ANOVA tests were emphasised over individual pairwise contrasts; *post hoc* comparisons (Fisher's Least Significant Difference, LSD) are reported but interpreted as exploratory because LSD does not itself correct for multiplicity; and a Bonferroni-adjusted reference threshold (*α* = .05/6 = .008) was applied when appraising each family of subgroup tests. Under this threshold most omnibus effects remained significant, whereas a small number that were only nominally significant (e.g., EN across education, *p* = .01; ASHN total across activity frequency, *p* = .008) are interpreted with corresponding caution. Throughout, effect sizes (Cohen's d, *η*^2^) rather than *p*-values alone guided substantive interpretation. In addition, to assess whether the observed subgroup differences persisted after mutual adjustment for overlapping characteristics, two univariate general linear models were estimated with PACES total and ASHN total as dependent variables; age was entered as a continuous covariate and education level, sports license status, marital status, and exercise frequency as fixed factors; each model comprised the demographic main effects together with their interactions (full factorial specification), and partial eta-squared was reported as the effect-size index for the adjusted main-effect tests. Statistical significance was set at *p* < .05.

## 3 Results

Table 1 summarises participant characteristics alongside available population comparators for adult women in Türkiye.

Table 2 indicates a positive and statistically significant correlation between PACES total score and ASHN total score (*r* = .28, *p* < .001), as well as the IN (*r* = .27, *p* < .001), PN (*r* = .27, *p* < .001), and MP (*r* = .14, *p* < .01) subscales. No significant relationship was observed between PACES and EN (*r* = .09, *p* > .05).

**Table 2:** Pearson correlations between PACES total and ASHN total and its subdimensions.

Variable	*n*	ASHN Total	IN	EN	PN	MP
PACES	397	.28**	.27**	.09	.27**	.14**

** *p* < .01.

n, number of participants; r, Pearson product-moment correlation coefficient. PACES, Physical Activity Enjoyment Scale; ASHN, Attitude Scale for Healthy Nutrition; IN, Information on Nutrition; EN, Emotion for Nutrition; PN, Positive Nutrition; MP, Malnutrition. The EN and MP subscales are reverse-scored, so higher values indicate a weaker affective pull toward energy-dense foods (EN) and fewer maladaptive eating patterns (MP), respectively; thus higher scores on all ASHN dimensions are health-favourable.

Table 3 shows the independent-samples *t*-test results comparing physical activity enjoyment and healthy nutrition attitudes by marital status. Married participants reported significantly higher ASHN total scores than single participants, t(395) = 4.22, *p* < .001, with a moderate effect size (Cohen's d = 0.42). Significant differences were also observed for the EN subscale, t(395) = 3.86, *p* < .001, d = 0.39 (moderate), and the MP subscale, t(395) = 4.30, *p* < .001, *d* = 0.43 (moderate), indicating more favourable scores among married women on these dimensions. In contrast, no significant differences were found between married and single participants for PACES total, IN, or PN (*p* > .05), and the corresponding effect sizes were negligible to small (*d* = −0.02 to 0.18). Overall, marital status was associated with specific dimensions of healthy nutrition attitudes, whereas enjoyment of physical activity did not differ meaningfully by marital status.

**Table 3:** Independent-samples *t*-test results for PACES and ASHN by marital status.

Variable	Married (*n* = 172)		Single (*n* = 225)		*t*	df	*p*	Cohen d
	M	SD	M	SD				
PACES Total	49.22	8.21	49.44	8.65	−0.26	395	.80	−0.02
ASHN Total	80.52	12.71	74.83	13.75	4.22	395	<.001	0.42
IN	20.93	4.82	20.82	4.21	0.24	395	.81	0.02
EN	19.90	6.09	17.60	5.68	3.86	395	<.001	0.39
PN	19.56	4.16	18.68	5.15	1.88	395	.06	0.18
MP	20.12	5.17	17.71	5.94	4.30	395	<.001	0.43

n, number of participants; M, mean; SD, standard deviation; df, degrees of freedom; t, independent-samples t statistic; d, Cohen's d (effect size). PACES, Physical Activity Enjoyment Scale; ASHN, Attitude Scale for Healthy Nutrition; IN, Information on Nutrition; EN, Emotion for Nutrition; PN, Positive Nutrition; MP, Malnutrition. *p* < .05 was considered statistically significant.

As shown in Table 4, a statistically significant difference by athletic license status was observed only for the EN subscale (*p* < .05). EN scores were higher among unlicensed participants than licensed participants, with a small effect size (Cohen's *d* = 0.20). No statistically significant differences were found for PACES total, ASHN total, IN, PN, or MP (*p* > .05), and effect sizes were negligible to small (*d* = 0.00–0.10). Overall, athletic license status showed a limited association confined to the EN dimension.

**Table 4:** Independent-samples *t*-test results for PACES and ASHN by athletic license status.

Variable	Licensed (*n* = 222)		Unlicensed (*n* = 175)		*t*	df	*p*	Cohen d
	M	SD	M	SD				
PACES Total	49.36	8.46	49.33	8.46	0.04	395	.96	0.00
ASHN Total	76.78	13.64	77.96	13.54	−0.85	395	.39	−0.08
IN	21.07	3.83	20.61	5.19	1.01	395	.31	0.10
EN	18.07	5.97	19.27	5.90	−1.99	395	.04	−0.20
PN	18.99	4.60	19.16	4.97	−0.35	395	.72	−0.03
MP	18.63	5.59	18.90	5.93	−0.46	395	.64	−0.04

n, number of participants; M, mean; SD, standard deviation; df, degrees of freedom; t, independent-samples t statistic; d, Cohen's d (effect size). PACES, Physical Activity Enjoyment Scale; ASHN, Attitude Scale for Healthy Nutrition; IN, Information on Nutrition; EN, Emotion for Nutrition; PN, Positive Nutrition; MP, Malnutrition. *p* < .05 was considered statistically significant.

Table 5 presents the one-way ANOVA results comparing physical activity enjoyment and healthy nutrition attitudes across educational attainment groups (high school, bachelor's, postgraduate). Significant group differences were found for PACES total scores (*F* = 8.88, *p* < .001, *η*^2^ = 0.04), indicating a small effect of education level on physical activity enjoyment. *post hoc* LSD comparisons showed that participants with a bachelor's degree and those with postgraduate education scored significantly higher on PACES than high school graduates (2-1; 3-1).

**Table 5:** ANOVA results for PACES and ASHN by educational attainment.

Variable	Group	*n*	M	SD	F	p/LSD	*η* ^2^
PACES Total	1. High School	100	46.33	10.57	8.88	<.001 (2-1; 3-1)	0.04
	2. Bachelor's Degree	233	50.36	7.29			
	3. Postgraduate	64	50.39	7.62			
	Total	397	49.35	8.45			
ASHN Total	1. High School	100	75.51	13.90	13.80	<.001 (3-1; 3-2)	0.06
	2. Bachelor's Degree	233	75.89	13.71			
	3. Postgraduate	64	85.21	9.44			
	Total	397	77.30	13.59			
IN	1. High School	100	19.46	5.57	7.58	<.001 (2-1; 3-1; 3-2)	0.03
	2. Bachelor's Degree	233	21.18	3.99			
	3. Postgraduate	64	21.93	3.77			
	Total	397	20.87	4.48			
EN	1. High School	100	19.39	6.18	4.51	.01 (2-1; 3-1; 3-2)	0.02
	2. Bachelor's Degree	233	17.87	6.26			
	3. Postgraduate	64	20.03	3.77			
	Total	397	18.60	5.96			
PN	1. High School	100	18.37	5.59	6.32	<.001 (3-1; 3-2)	0.03
	2. Bachelor's Degree	233	18.85	4.45			
	3. Postgraduate	64	20.92	4.00			
	Total	397	19.06	4.76			
MP	1. High School	100	18.29	5.72	15.96	<.001 (3-1; 3-2)	0.07
	2. Bachelor's Degree	233	17.97	5.97			
	3. Postgraduate	64	22.32	2.98			
	Total	397	18.75	5.74			

n, number of participants; M, mean; SD, standard deviation; F, ANOVA F ratio; LSD, Fisher's Least Significant Difference *post hoc* test (group contrasts coded by row number); η², eta squared (effect size). PACES, Physical Activity Enjoyment Scale; ASHN, Attitude Scale for Healthy Nutrition; IN, Information on Nutrition; EN, Emotion for Nutrition; PN, Positive Nutrition; MP, Malnutrition. *post hoc* comparisons are interpreted as exploratory; *p* < .05 was considered statistically significant.

Education level was also significantly associated with ASHN total scores (*F* = 13.80, *p* < .001, *η*^2^ = 0.06), reflecting a small-to-moderate effect. *post hoc* results indicated that postgraduate participants reported higher ASHN total scores than both high school and bachelor's groups (3-1; 3-2). Similarly, significant differences emerged for all ASHN subdimensions: IN (*F* = 7.58, *p* < .001, *η*^2^ = 0.03), EN (*F* = 4.51, *p* = .01, *η*^2^ = 0.02), PN (*F* = 6.32, *p* < .001, *η*^2^ = 0.03), and MP (*F* = 15.96, *p* < .001, *η*^2^ = 0.07). Effect sizes ranged from small (*η*^2^ = 0.02–0.03) to moderate for MP (*η*^2^ = 0.07), suggesting that education level was most strongly related to differences in maladaptive nutrition patterns. Overall, the pattern indicates progressively more favourable profiles-higher enjoyment of physical activity and more positive nutrition attitudes-at higher educational levels, with the most pronounced differences observed for postgraduate participants.

Table 6 reports one-way ANOVA results comparing physical activity enjoyment and healthy nutrition attitudes across physical activity frequency groups (none, 1–2 days/week, 3–4 days/week, ≥5 days/week). PACES total scores differed significantly by activity frequency (*F* = 9.46, *p* < .001, *η*^2^ = 0.06), indicating a small-to-moderate effect. *post hoc* LSD comparisons showed that participants reporting no physical activity had significantly lower PACES scores than those active 1–2 days/week, 3–4 days/week, and ≥5 days/week (2-1; 3-1; 4-1).

**Table 6:** ANOVA results for PACES and ASHN by physical activity frequency.

Variable	Group	n	M	SD	F	p/LSD	η^2^
PACES Total	1. None	89	45.39	10.19	9.46	<.001 (4-1; 3-1; 2-1)	0.06
	2. 1–2 days/week	141	49.95	8.09			
	3. 3–4 days/week	94	50.57	6.94			
	4. ≥5 days/week	73	51.45	7.06			
	Total	397	49.35	8.45			
ASHN Total	1. None	89	74.95	12.65	4.04	.008 (4-1; 4-2)	0.03
	2. 1–2 days/week	141	75.85	14.34			
	3. 3–4 days/week	94	78.44	13.47			
	4. ≥5 days/week	73	81.49	12.47			
	Total	397	77.30	13.59			
IN	1. None	89	20.83	4.38	4.91	<.001 (4-1; 4-2; 4-3)	0.03
	2. 1–2 days/week	141	20.15	4.98			
	3. 3–4 days/week	94	20.67	4.43			
	4. ≥5 days/week	73	22.57	3.10			
	Total	397	20.87	4.48			
EN	1. None	89	18.51	5.73	0.68	.55 (ns)	0.00
	2. 1–2 days/week	141	18.51	5.86			
	3. 3–4 days/week	94	19.29	6.13			
	4. ≥5 days/week	73	18.01	6.25			
	Total	397	18.60	5.96			
PN	1. None	89	17.88	4.99	5.72	<.001 (4-1; 4-2)	0.04
	2. 1–2 days/week	141	18.65	4.80			
	3. 3–4 days/week	94	19.45	4.69			
	4. ≥5 days/week	73	20.78	3.95			
	Total	397	19.06	4.76			
MP	1. None	89	17.71	5.78	2.54	.06 (ns)	0.01
	2. 1–2 days/week	141	18.52	5.87			
	3. 3–4 days/week	94	19.02	5.52			
	4. ≥5 days/week	73	20.13	5.52			
	Total	397	18.75	5.74			

ns = not significant. n, number of participants; M, mean; SD, standard deviation; F, ANOVA F ratio; LSD, Fisher's Least Significant Difference *post hoc* test (group contrasts coded by row number, e.g., 4-1 = ≥5 days/week vs. none); η^2^, eta squared. PACES, Physical Activity Enjoyment Scale; ASHN, Attitude Scale for Healthy Nutrition; IN, Information on Nutrition; EN, Emotion for Nutrition; PN, Positive Nutrition; MP, Malnutrition. *post hoc* comparisons are interpreted as exploratory.

ASHN total scores also differed significantly across frequency groups (*F* = 4.04, *p* = .008, *η*^2^ = 0.03; small effect). *post hoc* results indicated that women active ≥5 days/week scored higher than those reporting no activity and those active 1–2 days/week (4-1; 4-2). Similar significant differences were observed for the IN subscale (*F* = 4.91, *p* < .001, *η*^2^ = 0.03; small), with the ≥5 days/week group scoring higher than the none, 1–2 days/week, and 3–4 days/week groups (4-1; 4-2; 4-3). PN scores also varied significantly by activity frequency (*F* = 5.72, *p* < .001, *η*^2^ = 0.04; small), and *post hoc* tests indicated higher PN scores in the ≥5 days/week group compared with the none and 1–2 days/week groups (4-1; 4-2).

In contrast, no significant differences were found for EN (*F* = 0.68, *p* = .55, *η*^2^ = 0.00) or MP (*F* = 2.54, *p* = .06, *η*^2^ = 0.01), and the corresponding effects were negligible. Overall, higher physical activity frequency was associated with greater enjoyment of physical activity and more favourable overall nutrition attitudes, particularly in the knowledge- and behaviour-oriented dimensions (IN and PN), whereas emotionally oriented and maladaptive-pattern dimensions (EN and MP) did not vary meaningfully across frequency groups.

Table 7 presents Pearson correlation coefficients between participants’ age and the study variables. Age was positively and significantly correlated with ASHN total (*r* = .25, *p* < .01), EN (*r* = .28, *p* < .01), and MP (*r* = .23, *p* < .01), indicating that higher age was associated with more favourable overall healthy nutrition attitudes, particularly in EN and MP dimensions. In contrast, age was not significantly related to PACES total (*r* = −.02, *p* > .05), IN (*r* = .02, *p* > .05), or PN (*r* = .08, *p* > .05), suggesting that enjoyment of physical activity and the knowledge- and positive-behaviour nutrition dimensions did not vary meaningfully with age in this sample.

**Table 7:** Pearson correlations between participants’ age and PACES and ASHN.

Variable	*n*	PACES Total	ASHN Total	IN	EN	PN	MP
Age	397	−.02	.25**	.02	.28**	.08	.23**

** *p* < .01.

n, number of participants; r, Pearson product-moment correlation coefficient. PACES, Physical Activity Enjoyment Scale; ASHN, Attitude Scale for Healthy Nutrition; IN, Information on Nutrition; EN, Emotion for Nutrition; PN, Positive Nutrition; MP, Malnutrition.

Table 8 shows that the simple linear regression model predicting PACES total from ASHN total was statistically significant, F(1, 395) = 35.826, *p* < .001. ASHN total positively predicted PACES total (B = 0.17, SE = 0.03, *β* = .28, *t* = 5.98, *p* < .001), indicating that more favourable attitudes toward healthy nutrition were associated with greater enjoyment of physical activity. The model explained 8.3% of the variance in PACES scores (R^2^ = .083; Adj. R^2^ = .081). The Durbin-Watson value (2.025) indicated no evidence of autocorrelation in residuals.

**Table 8:** Simple linear regression predicting PACES total from ASHN total.

Predictor	B	SE B	*Β*	*t*	*p*
Constant	35.49	2.35	–	15.09	<.001
ASHN Total	0.17	0.03	0.28	5.98	<.001

Model summary: R = .288; R^2^ = .083; Adj. R^2^ = .081; F(1, 395) = 35.826, *p* < .001; Durbin-Watson = 2.025. B, unstandardised regression coefficient; SE B, standard error of B; *β*, standardised coefficient; t, t statistic; R^2^, coefficient of determination; PACES, Physical Activity Enjoyment Scale; ASHN, Attitude Scale for Healthy Nutrition.

To address the possibility that the bivariate subgroup differences reported above were confounded by overlapping demographic characteristics, two univariate general linear models were estimated in which age (continuous covariate), education level, sports license status, marital status, and exercise frequency were entered simultaneously, with PACES total (Model A) and ASHN total (Model B) as dependent variables (Table 9). Each full model (main effects plus their interactions) accounted for a moderate share of variance (PACES, R^2^ = .365, adjusted R^2^ = .286; ASHN, R^2^ = .289, adjusted R^2^ = .200; both *p* < .001), although the difference between R^2^ and adjusted R^2^ indicates that much of this was attributable to numerous interaction terms that were not individually reliable, so interpretation focuses on the adjusted main effects. In the model for physical activity enjoyment, exercise frequency (F(3, 352) = 8.39, *p* < .001, partial *η*^2^ = .067), sports license status (F(1, 352) = 14.05, *p* < .001, partial *η*^2^ = .038), and education level (F(2, 352) = 6.24, *p* = .002, partial *η*^2^ = .034) retained independent, small-to-moderate associations, whereas marital status was only weakly associated (F(1, 352) = 4.22, *p* = .041, partial *η*^2^ = .012) and age was unrelated (*p* = .793). In the model for healthy nutrition attitudes, education level (partial *η*^2^ = .039), exercise frequency (partial *η*^2^ = .044), and age (partial *η*^2^ = .033) showed independent associations (all *p* ≤ .001), and license status remained significant (*p* = .013, partial *η*^2^ = .018). Notably, the marital-status difference in ASHN that was apparent in the unadjusted comparison (Table 3) was substantially attenuated and no longer statistically significant once the other characteristics were controlled (F(1, 352) = 1.22, *p* = .270, partial *η*^2^ = .003), indicating that the higher nutrition-attitude scores of married women are largely accounted for by their co-occurring age and educational profile rather than by marital status *per se*. All adjusted effects remained small to moderate in magnitude, consistent with the modest effect sizes observed throughout.

**Table 9:** Covariate-adjusted associations of demographic and activity-related characteristics with PACES and ASHN total, estimated with univariate general linear models (*N* = 397).

Predictor	df	*F*	*p*	Partial η^2^
Model A. Dependent variable: PACES total				
Age	1	0.07	.793	.000
Education level	2	6.24	.002	.034
Sports license status	1	14.05	<.001	.038
Marital status	1	4.22	.041	.012
Exercise frequency	3	8.39	<.001	.067
Model B. Dependent variable: ASHN total				
Age	1	11.85	<.001	.033
Education level	2	7.12	<.001	.039
Sports license status	1	6.28	.013	.018
Marital status	1	1.22	.270	.003
Exercise frequency	3	5.43	.001	.044

Each column reports the unique effect of a predictor adjusted for all other terms in the same model. Age was entered as a continuous covariate; education level, sports license status, marital status, and exercise frequency were entered as fixed factors. Each model comprised the demographic main effects together with their interactions (full factorial specification; total model df = 44); the table reports the adjusted main-effect tests. Overall model fit: PACES total, F(44, 352) = 4.61, *p* < .001, R^2^ = .365, adjusted R^2^ = .286; ASHN total, F(44, 352) = 3.25, *p* < .001, R^2^ = .289, adjusted R^2^ = .200. The gap between R^2^ and adjusted R² reflects the inclusion of multiple higher-order interaction terms, few of which were individually significant; the main-effect pattern is therefore emphasised. *N* = 397; no covariate data were missing. df, degrees of freedom; F, F statistic; Partial η^2^, partial eta-squared (effect size); PACES, Physical Activity Enjoyment Scale; ASHN, Attitude Scale for Healthy Nutrition.

## 4 Discussion

The findings indicate that women who report greater enjoyment of physical activity also tend to hold more positive attitudes toward healthy nutrition, suggesting that these constructs may cluster within a shared motivational and self-regulatory profile rather than functioning as independent lifestyle domains. The association was strongest for the IN and PN dimensions, indicating that intrinsically rewarding movement experiences may co-occur with stronger nutrition knowledge and greater endorsement of health-supportive dietary behaviours (49, 50). A smaller association with MP further suggests that higher enjoyment may coincide with greater awareness of, and aversion to, unhealthy eating patterns. In contrast, the absence of a significant association with EN implies that affect-driven responses to food may depend on additional psychosocial determinants beyond enjoyment of physical activity.

Across demographic contexts, the pattern suggests that structural and behavioural resources may shape both constructs through overlapping pathways. Education level and physical activity frequency showed the most consistent differences, which may reflect differences in health literacy, access to supportive environments, routine stability, and repeated exposure that builds competence and intrinsic motivation, conditions that can simultaneously support enjoyment of movement and more favourable nutrition attitudes (51–53). The clearest gradients emerged in the IN and PN dimensions, reinforcing that cognitive-behavioural components of nutrition attitudes may be especially responsive to resource- and routine-related factors.

Marital status was associated with higher healthy nutrition attitudes (total, EN, and MP), which may reflect shared routines (e.g., meal planning), household responsibilities, and social support that can shape nutrition-related attitudes even when enjoyment of physical activity remains similar across partner-status groups (54, 55). However, this bivariate difference was substantially attenuated and no longer statistically significant once age, education level, license status, and activity frequency were adjusted for simultaneously, so it is most plausibly attributable to the co-occurring age and educational profile of married women rather than to marital status *per se*. Athlete license status showed a limited pattern (EN higher among unlicensed participants); because EN is reverse-scored, this indicates that unlicensed women reported a comparatively weaker affective pull toward energy-dense, palatable foods. This isolated difference should be interpreted cautiously; this difference may reflect unmeasured contextual or psychosocial factors (e.g., stress coping or food-related affect regulation) rather than sport involvement *per se* (56–58). Age showed modest associations with nutrition attitudes but not with enjoyment, suggesting that enjoyment of movement may be relatively stable across adulthood, whereas nutrition attitudes may shift with changing life experience and preventive orientation (2, 59).

Importantly, the overall pattern of results is consistent with the *a priori* hypothesis (H1) that affective engagement in physical activity (as reflected in PACES scores) and cognitive-emotional attitudes toward nutrition (as reflected in ASHN and its subscales) are interrelated dimensions of a health-oriented lifestyle. The observed correlations indicate that women who reported greater enjoyment of physical activity also tended to report more nutrition knowledge and more health-supportive dietary attitudes, together with fewer self-reported maladaptive eating patterns; because the design is cross-sectional, this co-occurrence does not establish that either domain shapes the other. This pattern is compatible with an integrated health-behaviour framework, within which interventions that promote physical activity may benefit from also addressing nutrition-related attitudes, and vice versa.

Because the observed associations were modest in magnitude (e.g., *r* = .28; R^2^ = 8.3% for the focal regression) and the subgroup effects were generally small (*η*^2^ = .02–.07), the following practical implications should be read as directions for intervention design rather than as evidence of large or clinically decisive effects. These integrated patterns indicate that health strategies may be more effective when they address both women's physical activity experiences and nutrition-related attitudes, while remaining responsive to subgroup contexts. Interventions that foster enjoyment, autonomy, and perceived competence in physical activity may support sustained engagement and align with more favourable nutrition-related attitudes. In parallel, nutrition education that strengthens practical skills (e.g., meal planning and label reading) may reinforce healthy dietary orientations, and tailoring delivery to demographic contexts may improve reach and impact.

### 4.1 Limitations

Several limitations should be acknowledged. The cross-sectional design precludes causal inference; self-report measures may introduce common-method and social-desirability bias; and convenience recruitment via online dissemination may limit generalisability beyond Turkish women with internet access. More specifically, convenience sampling through online channels is susceptible to self-selection bias: women who are more health-conscious, more digitally connected, or more interested in physical activity and nutrition may have been over-represented, which can inflate the observed associations and constrain generalisability to less-connected or less health-engaged women. Consistent with this, the sample diverged from national benchmarks (e.g., a younger mean age and a higher proportion with tertiary education than the adult female population of Türkiye), so the findings should be extrapolated only with caution. Physical activity was, moreover, captured with a single self-report item administered on one occasion; this item does not index intensity, duration, or domain of activity, is vulnerable to recall and social-desirability bias, and offers no indication of temporal stability, so objectively measured or multi-item activity assessment is warranted in future work. The demographic correlates of both constructs were re-examined in covariate-adjusted general linear models (Table 9), which indicated that several bivariate subgroup differences, most notably the marital-status difference in ASHN, did not persist after mutual adjustment; this strengthens confidence that the surviving associations (education, exercise frequency, and license status) are not merely artefacts of confounding. The focal ASHN-PACES association, however, was tested only with an unadjusted simple regression and was not re-estimated with these covariates entered alongside the predictor, so residual confounding of that specific coefficient cannot be excluded; a fully adjusted model entering sociodemographic and activity-related covariates together with ASHN would further isolate its unique contribution. The subgroup analyses also entailed multiple comparisons; although a Bonferroni-adjusted reference threshold was applied and effect sizes were emphasised, the *post hoc* LSD procedure is comparatively liberal, so the smaller subgroup differences in particular require replication. Finally, the associations and group differences were modest and the focal model explained only a small share of variance (R^2^ = 8.3%), indicating that the constructs examined capture only part of a broader determinant set and that their practical value should be appraised accordingly. In addition, unmeasured socioeconomic and contextual factors (e.g., income, work demands, access to facilities/healthy foods) may partly explain subgroup differences. Future research should use longitudinal designs to clarify directionality and apply theory-driven modelling (e.g., structural equation modelling) to test mediators and moderators. Including objective physical activity indicators and more comprehensive dietary measures, alongside richer socioeconomic and contextual variables, would strengthen inference and guide targeted interventions.

## 5 Conclusion

The findings indicate that women's enjoyment of physical activity and their attitudes toward healthy nutrition tend to co-occur, especially in domains reflecting nutrition knowledge and intentional healthy practices. This supports an integrated interpretation in which positive, autonomy-consistent engagement in physical activity aligns with broader health-oriented attitudes, whereas emotionally driven eating appears to be influenced by additional determinants not captured by enjoyment alone. Demographic differences, most consistently by education level and physical activity frequency, suggest that everyday opportunities and constraints shape these patterns and should be considered in interpretation and practice. Therefore, interventions may be more effective when they jointly promote enjoyable physical activity experiences and practical nutrition competencies, while being tailored to subgroup contexts to improve relevance and sustainability.

## Data Availability

The raw data supporting the conclusions of this article will be made available by the authors, without undue reservation.
