# Supplementary material for: Physical activity enjoyment and attitudes toward healthy nutrition in women: associations and demographic differences
Source: Front Glob Womens Health. 2026 Jul 8;7:1880236. doi: 10.3389/fgwh.2026.1880236 (PMC13388795; doi:10.3389/fgwh.2026.1880236)
Supplement: Supplementary file 1 [file Table1.docx]

**Supplementary File 1. STROBE Checklist (Cross-sectional Studies)**

Physical Activity Enjoyment and Attitudes Toward Healthy Nutrition in Women: Associations and Demographic Differences

| **Section** | **Item** | **Recommendation** | **Location in manuscript** |
| --- | --- | --- | --- |
| Title and abstract | 1a | Indicate the study's design with a commonly used term in the title or the abstract. | Abstract: Design; Methods |
| Title and abstract | 1b | Provide in the abstract an informative and balanced summary of what was done and what was found. | Abstract (all subheadings) |
| Introduction | 2 | Explain the scientific background and rationale for the investigation being reported. | Introduction (paragraphs 1-4) |
| Introduction | 3 | State specific objectives, including any prespecified hypotheses. | End of Introduction (aim statement) |
| Methods | 4 | Present key elements of study design early in the paper. | 2.1 Study Design |
| Methods | 5 | Describe the setting, locations, and relevant dates, including periods of recruitment, exposure, follow-up, and data collection. | 2.2 Participants (online recruitment across Türkiye; data collection April–June 2025; ethical approval Istanbul Aydın University, Decision No. 2025/3) |
| Methods | 6a | Give the eligibility criteria, and the sources and methods of selection of participants. | 2.2 Participants (eligibility and exclusions; convenience sampling) |
| Methods | 6b | For matched studies, give matching criteria and number of exposed and unexposed. (If applicable) | Not applicable (no matching). |
| Methods | 7 | Clearly define all outcomes, exposures, predictors, potential confounders, and effect modifiers. Give diagnostic criteria, if applicable. | 2.3 Data Collection Tools; 2.3.1 PACES; 2.3.2 ASHN; 2.3.3 Physical Activity Level; 2.4 Statistical Analysis |
| Methods | 8 | For each variable of interest, give sources of data and details of methods of assessment (measurement). Describe comparability of assessment methods if there is more than one group. | 2.3.1-2.3.3 (instrument descriptions and scoring) |
| Methods | 9 | Describe any efforts to address potential sources of bias. | 2.2 Participants (a priori safeguards: eligibility filtering, duplicate-entry and inattentive-response screening, mandatory electronic informed consent); 4.1 Limitations (common-method, social-desirability, and selection biases arising from self-report and online convenience sampling are acknowledged) |
| Methods | 10 | Explain how the study size was arrived at. | 2.2 Participants (final analytical N = 397; G*Power sensitivity analysis based on Fisher’s z transformation: 80% power, two-tailed α = .05, minimum detectable r ≈ .14) |
| Methods | 11 | Explain how quantitative variables were handled in the analyses. If applicable, describe which groupings were chosen and why. | 2.3.3 PA frequency (4-category grouping) and Table 1 note; 2.4 Statistical Analysis |
| Methods | 12a | Describe all statistical methods, including those used to control for confounding. | 2.4 Statistical Analysis |
| Methods | 12b | Describe any methods used to examine subgroups and interactions. | Subgroup comparisons: Tables 3-6 (t-tests/ANOVA). No interaction/moderation tests conducted. |
| Methods | 12c | Explain how missing data were addressed. | Complete-case analysis applied. The online platform required full completion of all items, and incomplete questionnaires were excluded prior to analysis (2.2 Participants); the analytical dataset (N = 397) was screened for missing values and no missingness was identified within retained cases (2.4 Statistical Analysis). |
| Methods | 12d | If applicable, describe analytical methods taking account of sampling strategy. | Not applicable (non-probability convenience sample). |
| Methods | 12e | Describe any sensitivity analyses. | Not applicable (no sensitivity analysis beyond sample-size/power sensitivity). |
| Results | 13a | Report numbers of individuals at each stage of study (e.g., numbers potentially eligible, examined for eligibility, confirmed eligible, included in the study, completing follow-up, and analysed). | 2.2 Participants (final analytical sample N = 397; Table 1). The online recruitment platform did not retain a verifiable denominator of respondents who initiated but did not complete the survey; therefore, the number potentially eligible, examined for eligibility, and excluded at each stage is not fully recoverable. This reporting limitation is acknowledged (4.1 Limitations). No flow diagram is included. |
| Results | 13b | Give reasons for non-participation at each stage. | Not available/not recorded for online convenience recruitment; incomplete questionnaires were excluded (2.2). |
| Results | 13c | Consider use of a flow diagram. | Not included. |
| Results | 14a | Give characteristics of study participants (e.g., demographic, clinical, social) and information on exposures and potential confounders. | Table 1 |
| Results | 14b | Indicate number of participants with missing data for each variable of interest. | No per-variable missingness in the analytical dataset: complete-case analysis on N = 397 because incomplete questionnaires were excluded prior to analysis (2.2 Participants) and the online platform enforced item-level completion. |
| Results | 15 | Report numbers of outcome events or summary measures. | Tables 2-8 (means/SDs, correlations, regression coefficients) |
| Results | 16a | Give unadjusted estimates and, if applicable, confounder-adjusted estimates and their precision (e.g., 95% CI). Make clear which confounders were adjusted for and why they were included. | Unadjusted estimates with 95% precision indices reported: Pearson correlations (Tables 2, 7), simple linear regression (Table 8), and group comparisons with effect sizes (Tables 3–6). Confounder-adjusted multivariable models were not estimated because subgroup analyses were descriptive and the regression model tested a single a priori predictor (ASHN total → PACES); the absence of multivariable adjustment is acknowledged as a limitation (4.1 Limitations). |
| Results | 16b | Report category boundaries when continuous variables were categorized. | 2.3.3 PA frequency categories; Table 1 note |
| Results | 16c | If relevant, consider translating estimates of relative risk into absolute risk for a meaningful time period. | Not applicable (non-risk outcomes; cross-sectional psychometric scores). |
| Results | 17 | Report other analyses done (e.g., analyses of subgroups and interactions, and sensitivity analyses). | Subgroup comparisons by marital status, athletic license, education, and physical activity frequency (Tables 3–6) with LSD post hoc tests for ANOVA (Tables 5, 6); Pearson correlations with age (Table 7); simple linear regression of ASHN total on PACES total (Table 8). No formal interaction, moderation, or sensitivity analyses were conducted. |
| Discussion | 18 | Summarise key results with reference to study objectives. | Discussion (opening paragraphs) |
| Discussion | 19 | Discuss limitations of the study, taking into account sources of potential bias or imprecision. Discuss both direction and magnitude of any potential bias. | 4.1 Limitations (cross-sectional design, self-report, common-method and social-desirability bias, convenience sampling); future-directions content addressed within the same subsection |
| Discussion | 20 | Give a cautious overall interpretation of results considering objectives, limitations, multiplicity of analyses, results from similar studies, and other relevant evidence. | Discussion (overall interpretation and integration with literature) |
| Discussion | 21 | Discuss the generalisability (external validity) of the study results. | 4.1 Limitations (generalisability explicitly addressed: findings apply to adult Turkish women with internet access recruited via online convenience sampling; cross-cultural and offline-population generalisability requires further study) |
| Other information | 22 | Give the source of funding and the role of the funders for the present study and, if applicable, for the original study on which the present article is based. | Funding statement in Declarations: “This research received no external funding.” No external funders had any role in study design, data collection, analysis, interpretation, or manuscript preparation. |

**Reference**

Von Elm, E., Altman, D. G., Egger, M., Pocock, S. J., Gøtzsche, P. C., & Vandenbroucke, J. P. (2007). The Strengthening the Reporting of Observational Studies in Epidemiology (STROBE) Statement: Guidelines for Reporting Observational Studies. *PLoS Medicine*, *4*(10), e296. https://doi.org/10.1371/journal.pmed.0040296
