# Supplementary material for: Physical activity enjoyment and attitudes toward healthy nutrition in women: associations and demographic differences
Source: Front Glob Womens Health. 2026 Jul 8;7:1880236. doi: 10.3389/fgwh.2026.1880236 (PMC13388795; doi:10.3389/fgwh.2026.1880236)
Supplement: Supplementary file 2 [file Table2.docx]

**Supplementary File 2. Measurement Instruments**

*Physical Activity Enjoyment Scale (PACES/FAKÖ) and Attitude Scale for Healthy Nutrition (ASHN/SBİTÖ)*

This supplementary file provides the item wording, response options, and scoring rules for the measurement instruments used in the study. The instruments are reproduced for transparency and peer-review purposes; any reuse should comply with the rights and conditions set by the original authors/publishers.

# 1. Physical Activity Enjoyment Scale (PACES) – Turkish form (FAKÖ)

Description: An 8-item, single-factor self-report scale assessing the extent to which individuals experience enjoyment during physical activity.

Response format: 7-point Likert-type scale (1 = Kesinlikle katılmıyorum / Strongly disagree; 7 = Kesinlikle katılıyorum / Strongly agree).

| **Item** | **PACES/FAKÖ item wording (Turkish)** |
| --- | --- |
| **1** | Fiziksel aktiviteleri zevkli buluyorum. |
| **2** | Fiziksel aktiviteler çok eğlencelidir. |
| **3** | Fiziksel aktiviteler hoştur. |
| **4** | Fiziksel aktiviteler canlandırıcıdır. |
| **5** | Fiziksel aktiviteler tatmin edicidir. |
| **6** | Fiziksel aktiviteler mutluluk vericidir. |
| **7** | Fiziksel aktiviteler harekete geçiricidir. |
| **8** | Fiziksel aktiviteler rahatlatıcıdır. |

*Scoring:* Compute either the sum score (range: 8–56) or the mean score (range: 1–7) across the 8 items. Higher scores indicate greater enjoyment of physical activity.

# 2. Attitude Scale for Healthy Nutrition (ASHN) – Turkish form (SBİTÖ)

*Description:* A 21-item scale with four subscales assessing attitudes toward healthy nutrition: Information on Nutrition (IN / Beslenme Hakkında Bilgi), Emotion for Nutrition (EN / Beslenmeye Yönelik Duygu), Positive Nutrition (PN / Olumlu Beslenme), and Malnutrition (MP / Kötü Beslenme).

*Response format:* 5-point Likert-type scale (1 = Kesinlikle Katılmıyorum / Strongly disagree; 5 = Kesinlikle Katılıyorum / Strongly agree).

*Reverse scoring:* Items 6–11 and 17–21 are negatively keyed and should be reverse-scored before computing subscale/total scores.

| **Item** | **ASHN/SBİTÖ item wording (Turkish)** |
| --- | --- |
| **1** | Sağlıklı beslenmenin yararlarını bilirim. |
| **2** | Hangi besinlerin protein içerdiğini bilirim. |
| **3** | Hangi besinlerin karbonhidrat içerdiğini bilirim. |
| **4** | Hangi besinlerin vitamin/mineral içerdiğini bilirim. |
| **5** | Sağlıklı besinlerin neler olduğunu bilirim. |
| **6** | Şekerli besinler (çikolata, kek, bisküvi, vb.) tükettiğimde mutlu olurum. |
| **7** | Fastfood ürünler (hamburger, pizza vb.) yemekten keyif alırım. |
| **8** | Şarküteri ürünleri (salam, sosis, sucuk, vb.) yemekten zevk alırım. |
| **9** | Yağda kızarmış besinlerin yemeyi severim. |
| **10** | Meyve tüketmekten hoşlanmam. |
| **11** | Şerbetli tatlıları (baklava, künefe vb.) tükettiğimde mutlu olurum. |
| **12** | Ana öğünleri (kahvaltı-öğle ve akşam yemeği) düzenli yerim. |
| **13** | Günde en az 1,5 lt su içerim. |
| **14** | Haftada en az 3 öğün sebze tüketirim. |
| **15** | Düzenli meyve tüketirim. |
| **16** | Her gün protein içeren besinler (et, süt, yumurta, vb.) yerim. |
| **17** | Ana öğünleri atlarım. |
| **18** | Her gün abur cubur (cips, çikolata, bisküvi, vb.) yerim. |
| **19** | Her gün asitli/gazlı içeceklerden en az 1 bardak içerim. |
| **20** | Ayaküstü beslenirim. |
| **21** | Ana öğünümü genellikle kek, bisküvi gibi gıdalarla geçiştiririm. |

*Subscale structure:* IN items 1–5; EN items 6–11; PN items 12–16; MP items 17–21.

*Scoring*: After reverse-scoring the negatively keyed items, compute subscale scores by summing the relevant items. A total ASHN score can be computed by summing all 21 items (range: 21–105). Higher scores indicate more favourable attitudes toward healthy nutrition.

# References

1. Özkurt, B., Küçükibiş, H. F., & Eskiler, E. (2022). Fiziksel aktivitelerden keyif alma ölçeği (FAKÖ): Türk kültürüne uyarlama, geçerlik ve güvenirlik çalışması. Anemon Muş Alparslan Üniversitesi Sosyal Bilimler Dergisi, 10(1), 21–37. doi:10.18506/anemon.976300
2. Tekkurşun Demir, G., & Cicioğlu, H. İ. (2019). Sağlıklı beslenmeye ilişkin tutum ölçeği (SBİTÖ): Geçerlik ve güvenirlik çalışması. Gaziantep Üniversitesi Spor Bilimleri Dergisi, 4(2), 256–274. doi:10.31680/gaunjss.559462
