# Supplementary material for: Physical activity enjoyment and attitudes toward healthy nutrition in women: associations and demographic differences
Source: Front Glob Womens Health. 2026 Jul 8;7:1880236. doi: 10.3389/fgwh.2026.1880236 (PMC13388795; doi:10.3389/fgwh.2026.1880236)
Supplement: Supplementary file 3 [file Table3.docx]

**Supplementary File 3. English Questionnaire and Survey Guide**

**A. Participant Information and Consent**

Please read the information below and indicate your consent to participate.

• I have read and understood the study information, and I voluntarily agree to participate. (Yes/No)

**B. Background Questions**

1) Age (years): ____

2) Marital status: (Married / Single)

3) Education level: (High school / Bachelor’s degree / Postgraduate)

4) Athletic license status: Do you currently hold an athletic license? (Yes/No)

5) Physical activity frequency: On average, how many days per week do you engage in physical activity?

☐ 0 days/week

☐ 1–2 days/week

☐ 3–4 days/week

☐ 5 or more days/week

**C. Physical Activity Enjoyment Scale (PACES) – 8-item Turkish version (English wording)**

Instructions: Please indicate how much you agree with each statement about physical activity.

Response options (7-point Likert): 1 = Strongly disagree, 2 = Disagree, 3 = Somewhat disagree, 4 = Neither agree nor disagree, 5 = Somewhat agree, 6 = Agree, 7 = Strongly agree.

1. I find physical activities enjoyable.

2. Physical activities are very fun.

3. Physical activities are pleasant.

4. Physical activities are invigorating.

5. Physical activities are satisfying.

6. Physical activities make me feel happy.

7. Physical activities are motivating/energizing.

8. Physical activities are relaxing.

**D. Attitudes toward Healthy Nutrition Scale (ASHN) – 21-item Turkish version (English wording)**

Instructions: Please indicate how much you agree with each statement about nutrition and eating habits.

Response options (5-point Likert): 1 = Strongly disagree, 2 = Disagree, 3 = Neither agree nor disagree, 4 = Agree, 5 = Strongly agree.

Note: Items 6–11 and 17–21 are negatively keyed in the Turkish form and should be reverse-scored before computing subscale/total scores.

1. I know the benefits of healthy eating.

2. I know which foods contain protein.

3. I know which foods contain carbohydrates.

4. I know which foods contain vitamins/minerals.

5. I know which foods are healthy.

6. I feel happy when I consume sugary foods (chocolate, cake, biscuits, etc.).

7. I enjoy eating fast food products (hamburgers, pizza, etc.).

8. I enjoy eating deli/processed meat products (salami, sausage, sucuk, etc.).

9. I like eating fried foods.

10. I do not like eating fruit.

11. I feel happy when I consume syrup-based desserts (baklava, künefe, etc.).

12. I eat main meals (breakfast, lunch, and dinner) regularly.

13. I drink at least 1.5 liters of water per day.

14. I consume vegetables at least 3 meals per week.

15. I consume fruit regularly.

16. I eat foods containing protein (meat, milk, eggs, etc.) every day.

17. I skip main meals.

18. I eat junk food (chips, chocolate, biscuits, etc.) every day.

19. I drink at least one glass of carbonated/sugary soft drinks every day.

20. I eat on the go (standing/fast).

21. I usually replace my main meal with foods such as cake or biscuits.

**References**

Özkurt, B., Küçükibiş, H. F., & Eskiler, E. (2022). Fiziksel aktivitelerden keyif alma ölçeği (FAKÖ): Türk kültürüne uyarlama, geçerlik ve güvenirlik çalışması. Anemon Muş Alparslan Üniversitesi Sosyal Bilimler Dergisi, 10(1), 21–37. doi:10.18506/anemon.976300

Tekkurşun Demir, G., & Cicioğlu, H. İ. (2019). Sağlıklı beslenmeye ilişkin tutum ölçeği (SBİTÖ): Geçerlik ve güvenirlik çalışması. Gaziantep Üniversitesi Spor Bilimleri Dergisi, 4(2), 256–274. doi:10.31680/gaunjss.559462
